# Multi-omics elucidation of *Lactiplantibacillus plantarum* NKK20 in preventing PCOS via the gut-ovary axis: SCFAs-mediated microbiota-metabolite-immune crosstalk

**DOI:** 10.3389/fnut.2025.1709581

**Published:** 2026-01-15

**Authors:** Hao Xu, Xinyu Liu, Wen Sun, Xueyun Dong, Xuehui Liu, Yunhan Xie, Jiayuan He, Asmaa Ali, Min Chen, Liang Wu, Jie Ma, Keke Shao

**Affiliations:** 1 Department of Laboratory Medicine, School of Medicine, Jiangsu University, Zhenjiang, China; 2 Department of Laboratory Medicine, Yancheng First Hospital Affiliated of Nanjing University Medical School, The Yancheng Clinical College of Xuzhou Medical University, The First People's Hospital of Yancheng, Yancheng, China; 3 Critical Care Medicine, Jurong Hospital Affiliated to Jiangsu University, Zhenjiang, China; 4 Health Testing Center, Zhenjiang Center for Disease Control and Prevention, Zhenjiang, China; 5 Department of Pulmonary Medicine, Abbassia Chest Hospital, EMOH, Cairo, Egypt; 6 Public Experiment and Service Center, Jiangsu University, Zhenjiang, China; 7 Molecular Medical Research Center, Yancheng Clinical Medical College of Jiangsu University, Yancheng, China

**Keywords:** gut microbiota, gut-ovary axis, *Lactiplantibacillus plantarum* NKK20, metabolomics, polycystic ovary syndrome, short-chain fatty acids

## Abstract

**Purpose:**

Polycystic ovary syndrome (PCOS) is a clinically prevalent endocrine and metabolic disorder characterized by gut microbial disturbances and chronic low-grade inflammatory responses.

**Methods:**

This study explores the therapeutic potential and mechanistic insights of *Lactiplantibacillus plantarum* NKK20 (LP) in a PCOS murine model established through high-fat diet (HFD) and letrozole co-induction. By integrating multi-omics profiling (16S rRNA sequencing and untargeted metabolomics) with histopathological evaluation, we systematically assessed LP-mediated modulations of gut microbiota composition, metabolic signatures, ovarian function, and intestinal barrier integrity.

**Results:**

The results demonstrated that LP administration effectively counteracted metabolic dysregulation in PCOS mice, mitigating body weight gain, ameliorating lipid abnormalities (reduced total cholesterol, triglycerides, and LDL-C alongside elevated HDL-C), and lowering fasting glucose levels. Hormonally, LP suppressed hyperandrogenism, as evidenced by decreased testosterone, while rebalancing inflammatory mediators through IL-10 upregulation and concomitant reduction of TNF-*α*, IL-6, IL-1β, and MCP-1. Ovarian histomorphology revealed attenuated follicular cysts and enhanced luteinization. Critically, LP restored intestinal homeostasis by (i) augmenting short-chain fatty acid (SCFA) production—particularly butyrate—(ii) fortifying the gut barrier via increased ZO-1 and occludin expression, and (iii) diminishing circulating endotoxin. Microbial sequencing identified enrichment of Bacteroidetes and Muribaculum following LP treatment. Serum metabolomics further uncovered LP-induced normalization of steroid hormone biosynthesis and glycerophospholipid metabolism, coinciding with elevated anti-inflammatory mediators such as 6a-prostaglandin I1.

**Conclusion:**

Collectively, these findings delineate a novel preventive axis through which LP inhibits PCOS progression — namely, via coordinated “gut microbiota–metabolite–ovarian” crosstalk involving SCFA-mediated barrier restoration, microbial ecology stabilization, and suppression of ovarian inflammatory onset. This work advances the translational rationale for probiotic-based strategies in PCOS prevention.

## Introduction

Polycystic ovary syndrome (PCOS), one of the most prevalent endocrine and metabolic disorders among reproductive-aged women, exhibits a global prevalence ranging from 6 to 20% (1, 2). Characterized clinically by hyperandrogenism, ovulatory dysfunction, and polycystic ovarian morphology, this syndrome frequently co-occurs with metabolic disturbances such as insulin resistance and obesity (3, 4). Emerging research has elucidated that PCOS pathogenesis is closely linked to a chronic low-grade inflammatory state triggered by gut microbial dysbiosis (5–7). The conceptualization of the gut-ovary axis has offered transformative insights into disease mechanisms, revealing how gut-derived microbial metabolites (e.g., short-chain fatty acids and bile acids) may modulate ovarian function and systemic glucose-lipid homeostasis through immunoregulatory and endocrine pathways (8–10). Despite these advances, current first-line PCOS treatments—including oral contraceptives and insulin-sensitizing agents—remain limited by inconsistent therapeutic responses and notable adverse effects during prolonged use, particularly beyond six months (11). These clinical constraints have kindled significant interest in developing novel therapeutic strategies targeting the restoration of gut microenvironmental homeostasis as a promising alternative approach (12).

Emerging research has increasingly highlighted the pivotal role of gut microbiota dysbiosis in the pathogenesis of PCOS. Clinical observations consistently demonstrate distinct alterations in the gut microbial ecosystem among PCOS patients, marked by diminished *α*-diversity indices, an imbalanced Bacteroidetes/Firmicutes ratio, and notably reduced biosynthesis of short-chain fatty acids (SCFAs), particularly butyrate (13, 14). Of translational significance, emerging evidence highlights the therapeutic potential of probiotics to boost gut-derived short-chain fatty acids (SCFAs), particularly butyrate, in PCOS intervention—building on preclinical studies showing that fecal microbiota transplantation (FMT) from healthy donors ameliorates sex hormone dysregulation and reverses polycystic ovarian pathology (15, 16). These benefits are partly mediated by butyrate, a microbial metabolite with pleiotropic effects, including GPCR activation to improve insulin sensitivity, NF-κB suppression to reduce ovarian inflammation, and gut-microbiota-host crosstalk modulation to restore metabolic homeostasis (17–19). However, critical gaps remain in understanding how probiotic-induced SCFA elevation, specifically butyrate, orchestrates these effects, warranting mechanistic studies to optimize microbiota-targeted therapies for PCOS.

Among various microbiota-targeted interventions, probiotics have garnered increasing attention due to their polypharmacological potential, with *Lactiplantibacillus plantarum* (LP) emerging as a particularly promising candidate (20). This commensal strain, originally isolated from healthy human intestinal microbiota, demonstrates pronounced metabolic-modulating and anti-inflammatory properties (21). Our preliminary investigations with *Lactiplantibacillus plantarum* NKK20 (LP) revealed its dual capacity to enhance intestinal butyrate production and remodel bile acid metabolism through the farnesoid X receptor (FXR)-dependent pathway, effectively attenuating hyperactivation of the NF-κB/MAPK signaling cascade (21, 22). These mechanistic underpinnings correlate with its demonstrated therapeutic efficacy in preclinical models of metabolic disorders, including non-alcoholic fatty liver disease and diabetic kidney disease (DKD).

Extending beyond these findings, subsequent research has elucidated that LP exerts additional protective effects by (i) activating the Nrf2/HO-1 antioxidant axis to mitigate oxidative damage and (ii) reciprocally modulating gut microbial composition—selectively enriching beneficial taxa (e.g., *Akkermansia*) while suppressing potential pathobionts like *Proteobacteria*—thereby reinforcing intestinal barrier integrity (23–25). Crucially, these multimodal actions exhibit striking convergence with core PCOS pathophysiological hallmarks, including insulin resistance and low-grade inflammation (26). We therefore postulate that LP may orchestrate therapeutic effects in PCOS through integrated modulation of the “gut microbiota-metabolite-immune” axis, a hypothesis warranting systematic experimental validation.

Employing an integrative multi-omics approach, this investigation systematically evaluated the therapeutic efficacy and mechanistic insights of LP (*L. plantarum* NKK20) in a well-characterized PCOS murine model. State-of-the-art high-resolution metabolomics enabled comprehensive profiling of dynamic serum metabolite alterations, while 16S rRNA gene analysis provided multi-dimensional assessments of gut microbiota restructuring through *α*- and *β*-diversity metrics, with particular emphasis on phylogenetic shifts at the phylum and genus levels. The experimental design was strategically devised to delineate LP-mediated restoration of gut microbial dysbiosis in PCOS while elucidating its therapeutic modulation of core pathological features via the “microbiota-metabolite-ovary” axis. These findings are anticipated to establish a robust conceptual framework and preclinical evidence base for developing precision microbiota-targeted interventions against PCOS.

## Materials and methods

### Animal model establishment

Female C57BL/6 mice (8 weeks old) were obtained from Phenok Biosciences (Shanghai, China; Production license No.: SCXK(Hu)2023–0008) and housed in specific pathogen-free (SPF) conditions at the Laboratory Animal Center of Jiangsu University under controlled environmental parameters (22 ± 2 °C, 50–60% humidity, 12/12 h light/dark cycle) with ad libitum access to food and water. All experimental protocols were approved by the Institutional Animal Care and Use Committee of Jiangsu University (Approval No.: UJS-IACUC-AP-2024030032). The strain *L. plantarum* NKK20 was provided by the Zhenjiang Tianyi Biotechnology Research Institute (Zhenjiang, China). The animals were randomly assigned to three experimental groups (n = 6/group): normal control (NC), polycystic ovary syndrome (PCOS), and *L. plantarum* NKK20-treated (LP) groups. While NC group received standard chow, both PCOS and LP groups were maintained on a 60% high-fat diet for 4 weeks to induce metabolic dysfunction (27), followed by a 21-day oral administration of letrozole (1 mg/kg/day, Sigma-Aldrich; suspended in 0.5% carboxmethylcellulose sodium solution) to establish PCOS phenotype (28). Successful model induction was verified through daily vaginal cytology showing prolonged cornification (>10 consecutive days) with disrupted estrous cyclicity alongside hallmark endocrine alterations. Serological analyses confirmed that before the intervention, model mice exhibited significantly elevated serum testosterone (T) levels, and an increased luteinizing hormone to follicle-stimulating hormone (LH/FSH) ratio, compared to the control group fed a regular diet (*P*<0.05). These parameters collectively confirm the establishment of a PCOS-like hyperandrogenic and anovulatory phenotype prior to LP intervention.

The therapeutic intervention commenced concomitantly with high-fat feeding in the LP group, receiving daily probiotic supplementation (1 × 10^7^ CFU *L. plantarum* NKK20 suspended in PBS). Upon completing the experimental period, euthanasia was performed via intraperitoneal injection of urethane (700 mg/kg, Sigma-Aldrich) following a 6-h fasting period to ensure optimal drug absorption. After injection, mice were individually placed in separate cages lined with soft bedding and monitored continuously for loss of vital signs, including the absence of righting reflex, cessation of heartbeat, and respiratory arrest. To ensure death and minimize potential distress, cervical dislocation was subsequently performed as an adjunctive method. Final confirmation of death was based on the absence of all vital signs, including no detectable heartbeat, irreversible cessation of respiration. All procedures were conducted in accordance with institutional animal welfare guidelines. Biological specimens including serum samples (for hormonal profiling and metabolomics), colonic contents (for 16S rRNA sequencing), and ovarian tissues were collected for subsequent analyses. Fixed ovarian and colonic specimens (4% paraformaldehyde, paraffin-embedded) were subjected to histological and immunohistochemical examinations.

### Hormonal and inflammatory marker analyses

Serum concentrations of estradiol (E2), testosterone (T), luteinizing hormone (LH), and follicle-stimulating hormone (FSH) were quantified using commercial enzyme-linked immunosorbent assay (ELISA) kits (Meimian Biotechnology, Jiangsu, China) following the manufacturer’s protocols. For ovarian inflammatory markers, total RNA extraction was performed using RNA-easy Isolation Reagent (Vazyme, Nanjing, China), followed by first-strand cDNA synthesis with HiScript III Reverse Transcriptase (+gDNA wiper, Vazyme). Quantitative real-time PCR amplification was conducted on a Bio-Rad CFX96 system (Hercules, CA, USA) under standardized thermal cycling conditions: initial denaturation at 95 °C for 30 s, followed by 40 cycles of 95 °C for 5 s with annealing/extension temperatures optimized for each primer set (sequences provided in Supplementary Table S1). Relative mRNA expression levels were normalized to housekeeping genes and calculated using the comparative threshold cycle (2^−ΔΔCt^) method.

### Analysis of intestinal barrier integrity

Serum endotoxin levels were measured using a chromogenic limulus amebocyte lysate (LAL) assay (Xiamen Bioendo Technology, China; Cat# H245-1). After diluting serum samples 1:10 in endotoxin-free water, thermal pretreatment was performed at 70 °C for 10 min to deactivate interfering substances, followed by centrifugation (Beckman Coulter, Brea, CA, USA. 3,000 × g, 4 °C, 10 min) to obtain clear supernatants. A standard curve ranging from 0.01 to 1.0 EU/mL was prepared as per the manufacturer’s instructions. Subsequently, diluted samples or standards (50 μL) were mixed with an equal volume of LAL reagent in pyrogen-free tubes and incubated at 37 °C for 30 min. The reaction was terminated by adding 100 μL chromogenic substrate (p-nitroaniline), and absorbance was measured at 405 nm to determine endotoxin concentrations, and the mixture was incubated at 37 °C for an additional 6 min to allow for color development. Absorbance was then measured at 405 nm using a microplate reader (BioTek Synergy HT, Agilent, USA). Endotoxin concentrations in the samples were calculated by interpolating their absorbance values against the established standard curve. Intestinal tight junction proteins ZO-1 and Occludin were evaluated via immunohistochemistry (IHC). Colon tissues were fixed in 4% paraformaldehyde, paraffin-embedded, and sectioned (4 μm). Following deparaffinization and rehydration, antigen retrieval was conducted in sodium citrate buffer (pH 6.0, 95 °C, 15 min). Endogenous peroxidase activity was quenched by 3% H₂O₂ treatment, followed by blocking with 5% BSA for 30 min at room temperature. Tissue sections were incubated with primary antibodies against ZO-1 (Proteintech, China; 1:200) or Occludin (Proteintech, 1:150) overnight at 4 °C, followed by incubation with horseradish peroxidase (HRP)-conjugated secondary antibody (Beyotime, China; 1:500) for 1 h at room temperature. Diaminobenzidine (DAB, Beyotime, China) was used for chromogenic development, and nuclei were counterstained with hematoxylin before mounting in neutral resin. Image intensity analysis was performed using Image-Pro Plus 6.0 software (Media Cybernetics, Inc., Rockville, MD, USA; Version 6.0) to quantify protein expression levels.

### Gut microbiota 16S rRNA sequencing and bioinformatics analysis

Genomic DNA from murine colonic contents was isolated using a commercial extraction kit (TIANGEN, Beijing, China), followed by targeted amplification and sequencing of the 16S rRNA V3-V4 hypervariable region conducted by Wekemo Tech Group (Shenzhen, China). The raw sequencing data were processed through the cloud-based Wekemo Bioincloud platform (https://www.bioincloud.tech/) for comprehensive bioinformatic evaluation. Microbial community characteristics were assessed through multiple analytical approaches: *α*-diversity indices (Chao1, Shannon, and Simpson) quantified taxonomic richness and evenness; *β*-diversity patterns visualized via principal component analysis (PCA) revealed compositional clustering among experimental groups; and hierarchical clustering heatmaps illustrated differential abundance profiles of operational taxonomic units (OTUs) influenced by LP treatment.

### Untargeted metabolomics profiling and short-chain fatty acid quantification

For metabolite extraction, 100 μL of serum samples were mixed with 400 μL of ice-cold methanol (chromatography grade, Sigma-Aldrich, St. Louis, MO, USA) containing 0.1% formic acid (chromatography grade, Sigma-Aldrich). The mixture was homogenized by vortexing for 30 s, followed by 10 min of ice-bath ultrasonication. After centrifugation at 12,000 × g for 15 min (4 °C), the supernatants were filtered through 0.22 μm membranes prior to LC–MS analysis performed by Wekemo Tech Group (Shenzhen, China). Metabolic profiling was conducted using an Agilent 1,290 Infinity II UHPLC system (Agilent Technologies, Santa Clara, CA, USA) coupled with a 6,545 Q-TOF mass spectrometer (Agilent Technologies) equipped with an electrospray ionization (ESI) source. Separation was achieved on a HILIC column (2.1 × 100 mm, 1.7 μm) maintained at 40 °C with a 0.3 mL/min flow rate. The mobile phases consisted of aqueous 0.1% formic acid (A) and 0.1% formic acid in acetonitrile (B), employing the following gradient: 5–20% B from 0–2 min, 20–95% B through 2–10 min, 95% B for 10–12 min, with re-equilibration to initial conditions by 15 min. MS parameters included positive/negative ion switching mode, 300 °C drying gas temperature, 10 L/min nebulizer flow, 40 psi capillary pressure, and m/z 50–1,000 scan range with 20/40 eV stepped collision energy for MS/MS fragmentation. Raw LC–MS data were processed using Progenesis QI software (version 3.0; Waters Corporation, Milford, MA, USA) for feature extraction, retention time alignment, and intensity normalization. Metabolites were considered differentially abundant when meeting both VIP > 1 (from OPLS-DA models) and *p* < 0.05 (Student’s *t*-test). Compound identification was performed via mass accuracy (<5 ppm) and MS/MS spectral matching against HMDB (Human Metabolome Database, https://hmdb.ca), METLIN (https://metlin.scripps.edu), and LipidMaps databases (Lipid Maps Structure Database, https://www.lipidmaps.org), with critical identifications confirmed using authentic standards. Metabolic pathway enrichment analysis was executed through MetaboAnalyst 5.0 (https://www.metaboanalyst.ca) using KEGG (Kyoto Encyclopedia of Genes and Genomes; https://www.genome.jp/kegg/) reference pathways with FDR < 0.05 significance threshold.

The quantification of short-chain fatty acids (SCFAs) was performed in-house following the approach reported by He et al. (29), with modifications. Briefly, 100 μL of serum was combined with 300 μL of acetonitrile containing 1% formic acid, vortexed for 1 min, and centrifuged (12,000 rpm, 10 min, 4 °C). The supernatant was passed through a 0.22-μm PTFE membrane filter before injection. Chromatographic separation was achieved using an ACQUITY UPLC HSS T3 column (2.1 × 100 mm, 1.8 μm) maintained at 40 °C. The mobile phases consisted of 0.1% formic acid in water (A) and 0.1% formic acid in acetonitrile (B), delivered at a flow rate of 0.3 mL/min. The gradient elution protocol was as follows: initial 5% B (0 min), ramped to 30% B by 2 min, increased to 95% B at 4 min, held until 5 min, followed by immediate return to 5% B (5.1 min), and re-equilibration until 7 min. The injection volume was 2 μL. Mass spectrometric detection was performed in negative-ion electrospray ionization (ESI) mode with the following parameters: capillary voltage, 2.5 kV; ion source temperature, 150 °C; desolvation temperature, 500 °C; desolvation and cone gas flows, 1,000 and 150 L/h, respectively. Quantification was conducted in multiple reaction monitoring (MRM) mode. Authenticated standards for acetate, propionate, and butyrate (purity≥98%) were obtained from Sigma-Aldrich (USA).

### Statistical analysis

Statistical evaluation was conducted using SPSS 20.0 (IBM Corp., Armonk, NY, USA). Continuous variables were presented as mean ± standard deviation. For between-group comparisons, one-way analysis of variance (ANOVA) was applied when data met assumptions of normality and homogeneity of variance, with Tukey’s post-hoc test employed for multiple comparisons. A probability threshold of *p* < 0.05 was considered statistically significant. Data visualization was performed using GraphPad Prism 9.0 (GraphPad Software, San Diego, CA, USA) in combination with the Bioincloud online platform (https://bioincloud.tech/).

## Results

### Body weight, fasting blood glucose and lipid profiles in experimental mice

**Figure 1:**
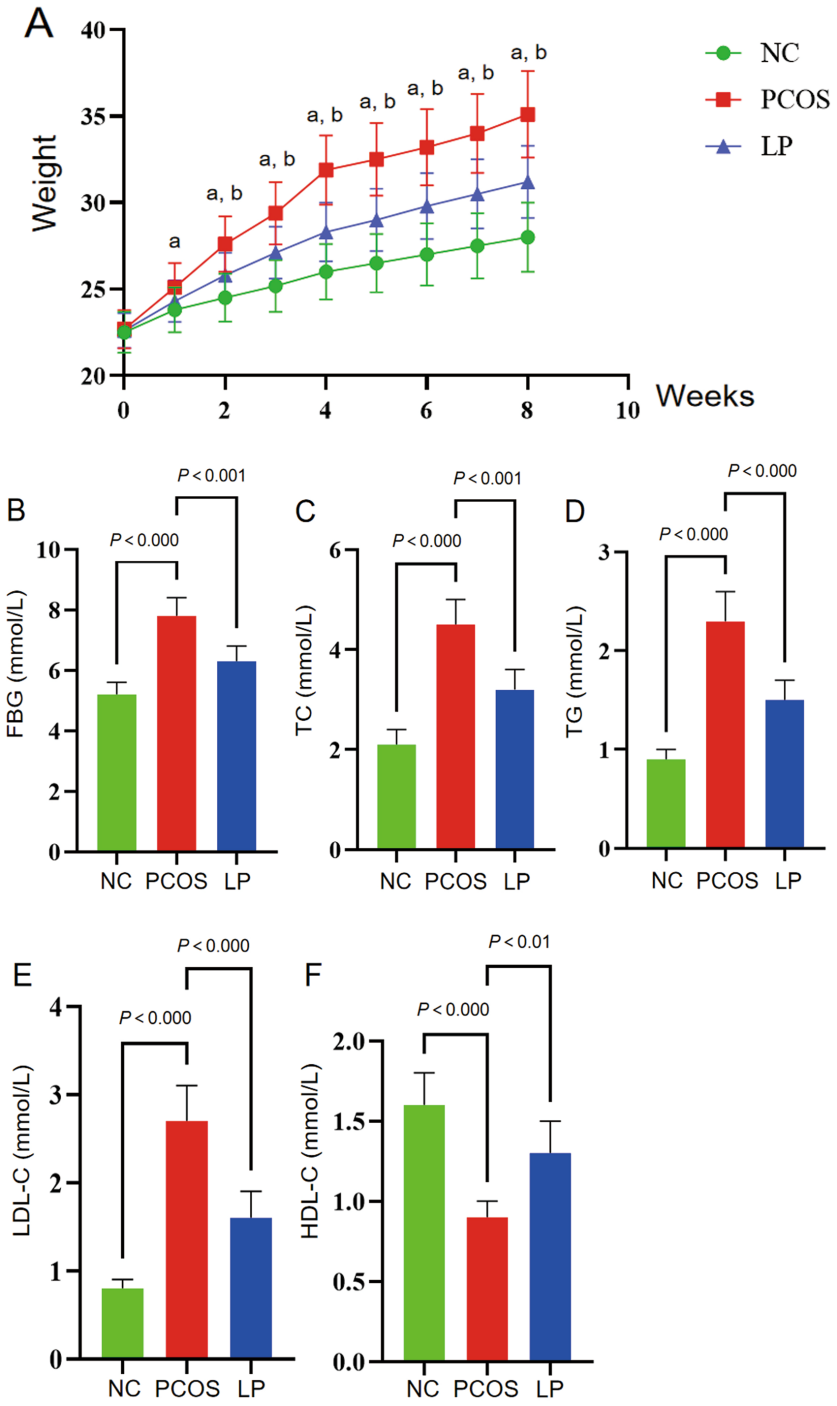
Body weight and serum biochemical parameters in experimental mice (*n* = 6). The figure panels illustrate: **(A)** Longitudinal body weight measurements, **(B)** fasting blood glucose (FBG), **(C)** total cholesterol (TC), **(D)** triglycerides (TG), **(E)** low-density lipoprotein cholesterol (LDL-C), and **(F)** high-density lipoprotein cholesterol (HDL-C). Statistical annotations indicate significant differences at *p* < 0.05 (a: versus NC group; b: versus PCOS group).

During the first week of high-fat diet (HFD) feeding, PCOS mice exhibited significantly higher body weight compared to NC animals (*p* < 0.05), with progressive weight gain observed throughout the study period. Letrozole administration did not alter this weight trajectory in PCOS mice. However, mice treated with LP (LP group) in combination with HFD showed attenuated weight gain relative to PCOS animals by the third week (*p* < 0.05), maintaining this suppression until study termination (Figure 1A). Terminal metabolic analyses revealed pronounced dyslipidemia in PCOS mice versus NC controls, as evidenced by elevated fasting blood glucose (FBG), total cholesterol (TC), triglycerides (TG), and low-density lipoprotein cholesterol (LDL-C) concentrations, coupled with reduced high-density lipoprotein cholesterol (HDL-C) levels (*p* < 0.05). Conversely, LP intervention substantially mitigated these alterations, demonstrating lower FBG, TC, TG, and LDL-C alongside increased HDL-C compared to untreated PCOS mice (*p* < 0.05) (Figures 1B–E).

### Serum sex hormone levels and ovarian inflammatory/damage markers in mice

**Figure 2:**
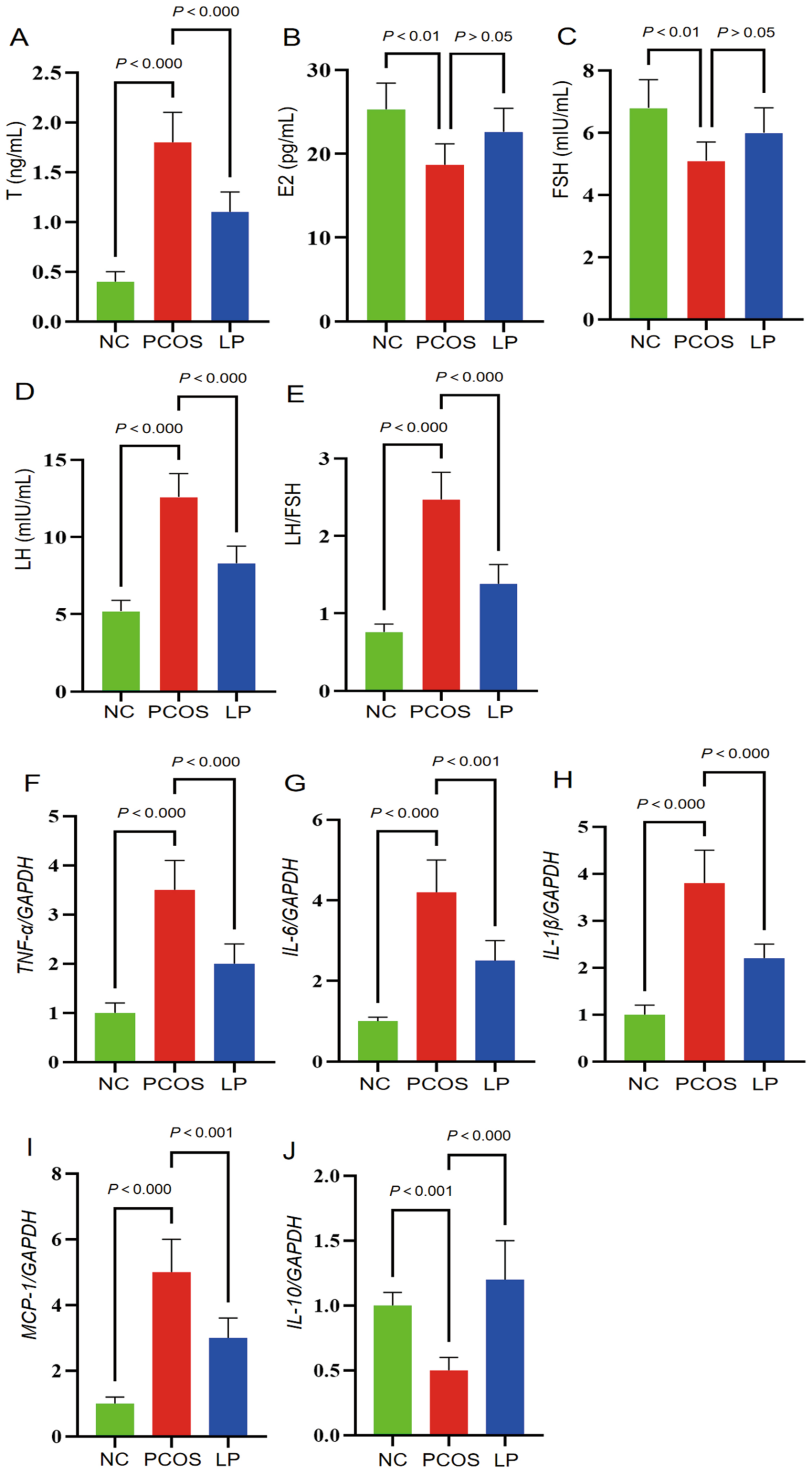
Serum sex hormone profiles and ovarian inflammatory markers in experimental mice (*n* = 6). The figure panels display: **(A)** Testosterone (T) concentrations, **(B)** estradiol (E2) levels, **(C)** follicle-stimulating hormone (FSH), **(D)** luteinizing hormone (LH), **(E)** LH/FSH ratio, **(F)** *TNF-α/GAPDH*, **(G)** *IL-6/GAPDH*, **(H)** *IL-1β/GAPDH*, **(I)** *MCP-1/GAPDH*, and **(J)** *IL-10/GAPDH*.

Serum sex hormone levels, including estradiol (E2), testosterone (T), luteinizing hormone (LH), and follicle-stimulating hormone (FSH), were quantified by ELISA, with the LH/FSH ratio subsequently calculated (Figures 2A–E). PCOS mice exhibited significantly elevated T, LH, and LH/FSH levels alongside reduced E2 and FSH concentrations compared to NC animals (*p* < 0.05). LP treatment effectively attenuated these hormonal imbalances, significantly lowering T, LH, and LH/FSH (*p* < 0.05), though E2 and FSH levels remained statistically unchanged relative to untreated PCOS mice (*p* > 0.05). Ovarian inflammatory cytokine expression was assessed *via* qRT-PCR (Figures 2F–J). PCOS animals displayed marked upregulation of proinflammatory mediators (TNF-*α*, IL-6, IL-1β, MCP-1) and suppression of the anti-inflammatory cytokine IL-10 versus NC controls (*p* < 0.05). LP administration mitigated this inflammatory response, significantly decreasing proinflammatory cytokine levels while restoring IL-10 expression (*p* < 0.05).

**Figure 3:**
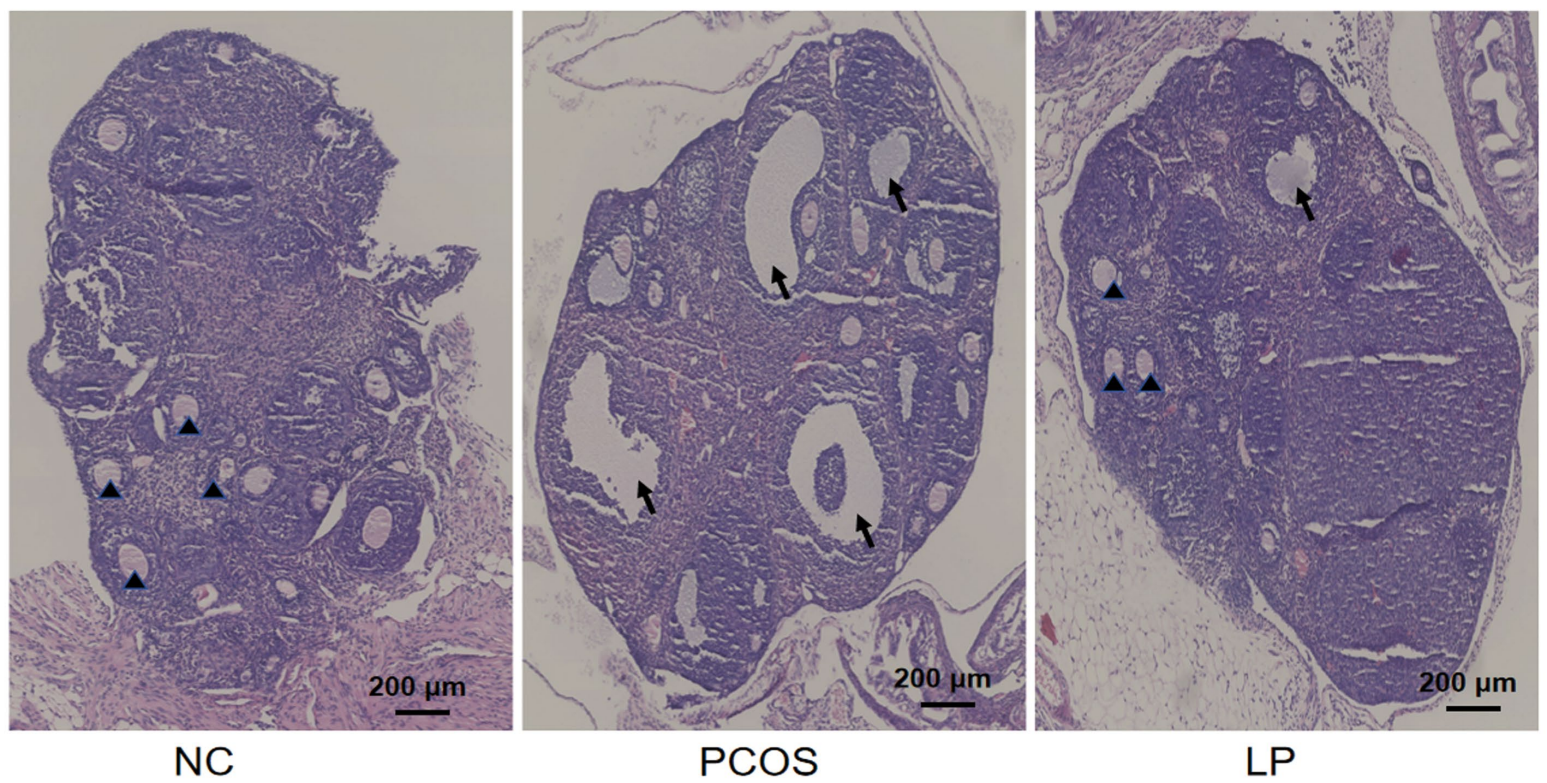
HE staining showing ovarian tissue damage in mice. ↑: Cystic follicle, ▲: corpus luteum.

Ovarian histomorphology evaluated by H&E staining (Figure 3) revealed striking structural differences. NC ovaries contained numerous healthy follicles with distinct granulosa cell layering, minimal atresia or vacuolation, normally distributed corpora lutea, and an absence of stromal hyperplasia, fibrosis, or inflammatory infiltrates. PCOS mice exhibited prominent cystic follicles, dilated antral follicles, extensive follicular atresia, sparse corpora lutea, and pronounced stromal cell proliferation. LP intervention partially reversed these pathologies, reducing cystic follicle prevalence, attenuating follicular atresia, promoting nascent corpus luteum formation, and alleviating stromal hyperplasia and inflammatory infiltration.

### LP ameliorates intestinal barrier dysfunction and systemic endotoxemia in PCOS mice

**Figure 4:**
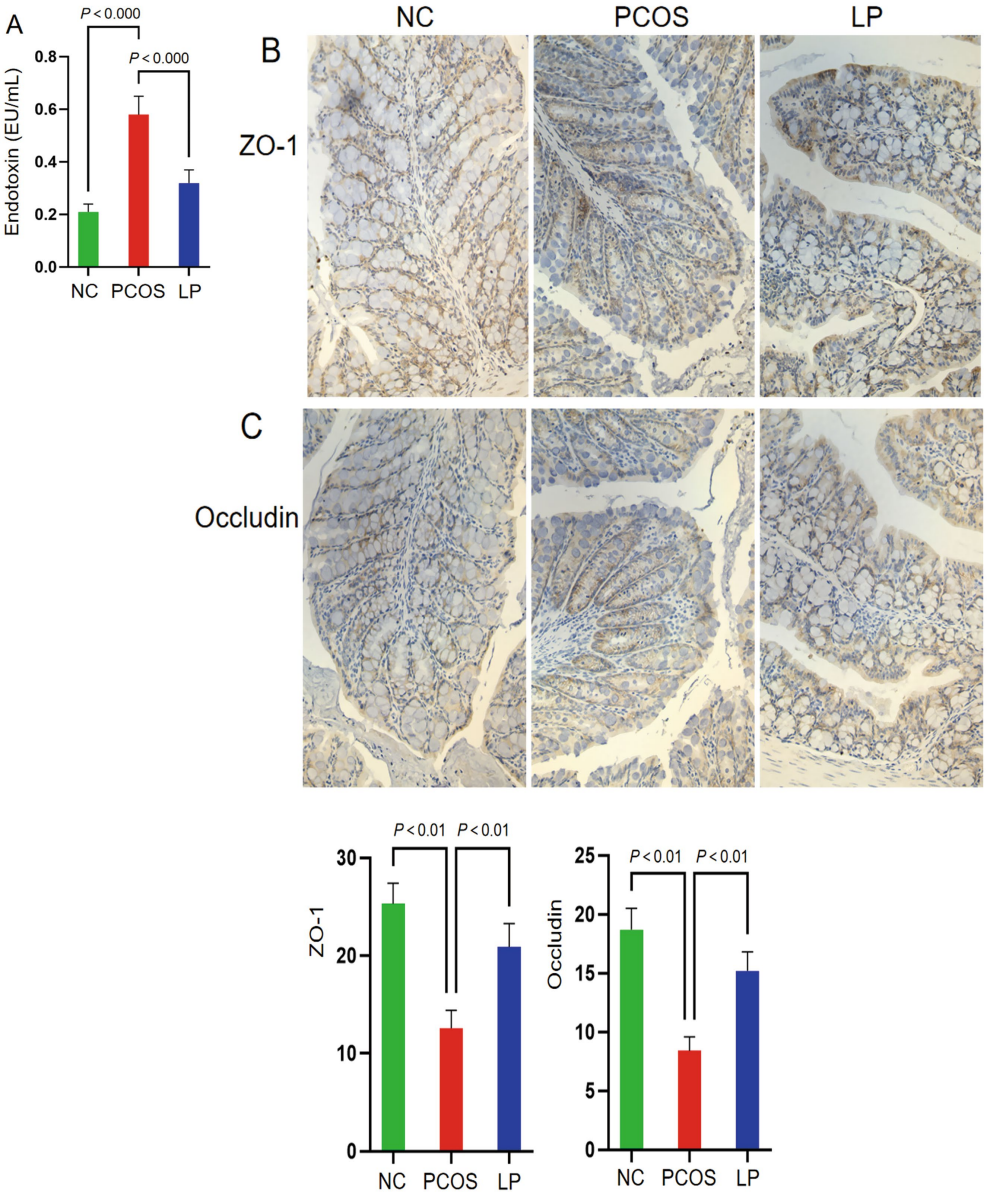
Serum endotoxin levels and intestinal mucosal expression of ZO-1 and Occludin in mice (*n* = 3). **(A)** Serum endotoxin levels; **(B)** ZO-1 expression; **(C)** Occludin.

PCOS mice exhibited significantly elevated serum endotoxin levels compared to NC controls (*p* < 0.05), demonstrating that high-fat diet and letrozole induction compromised intestinal permeability and induced endotoxemia. LP administration substantially attenuated this effect, with treated animals showing markedly lower circulating endotoxins than untreated PCOS counterparts (*p* < 0.05), indicating enhanced intestinal barrier integrity (Figure 4A). Immunohistochemical analysis revealed continuous, sharply defined ZO-1 and occludin staining along colonic mucosal cell borders in NC animals. PCOS mice displayed significantly diminished expression of these tight junction proteins (*p* < 0.05), exhibiting discontinuous immunoreactivity patterns consistent with epithelial barrier disruption. While LP intervention partially restored ZO-1 and occludin localization compared to PCOS mice, expression levels remained below those observed in NC controls (*p* < 0.05) (Figures 4B,C).

### Influence of LP on gut microbiota dysbiosis and restoration of SCFAs production in PCOS mice

**Figure 5:**
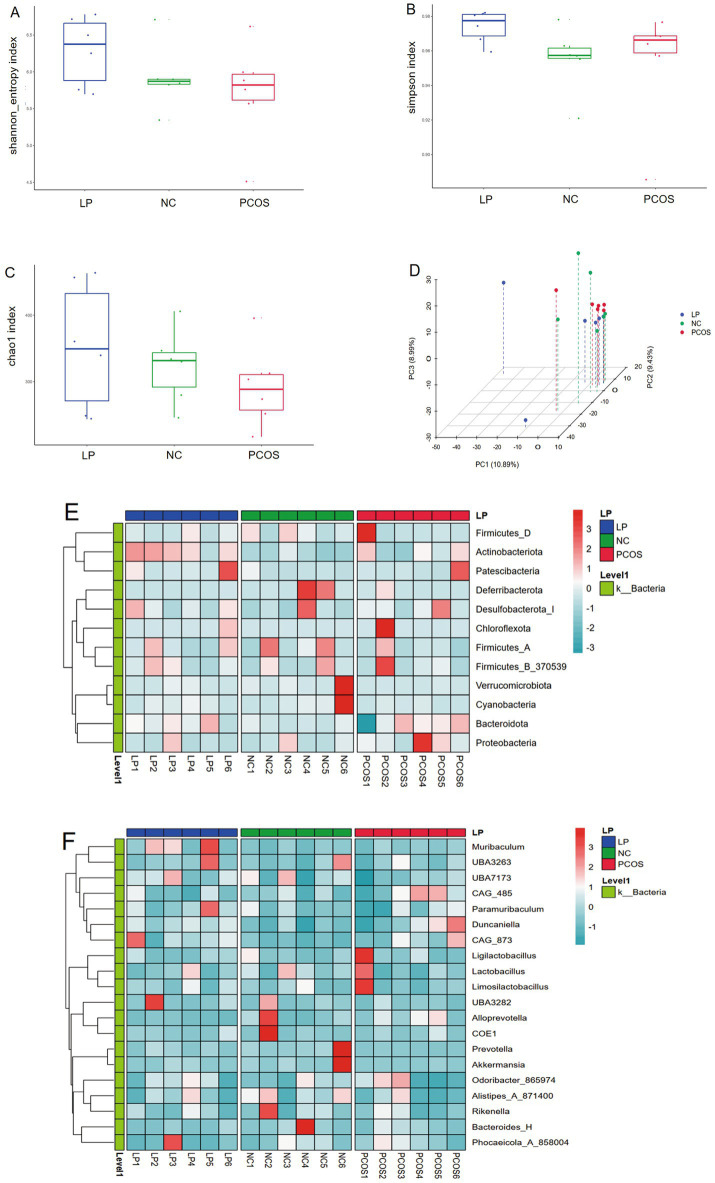
Gut microbiome profiling by 16S rRNA sequencing (*n* = 6). **(A)** Shannon diversity index, **(B)** Simpson diversity metrics, **(C)** Chao1 richness estimates, **(D)** Principal coordinate analysis of β-diversity, **(E)** Heatmap representation of phylum-level taxonomic composition, **(F)** Genus-level relative abundance heatmap.

Microbial community profiling via 16S rRNA sequencing revealed no significant intergroup differences in *α*-diversity indices (Shannon, Simpson, and Chao1) among NC, PCOS, and LP-treated mice (Figures 5A–C). However, β-diversity analysis demonstrated distinct clustering of LP-treated samples separate from PCOS controls (Figure 5D), indicating partial microbial community restoration following LP intervention. Taxonomic evaluation identified significant increases in *Actinobacteriota* and *Proteobacteria* at the phylum level (Figure 5E), along with elevated *Muribaculum* and *UBA7173* abundance at the genus level in LP-administered mice compared to PCOS counterparts (Figure 5F).

**Figure 6:**
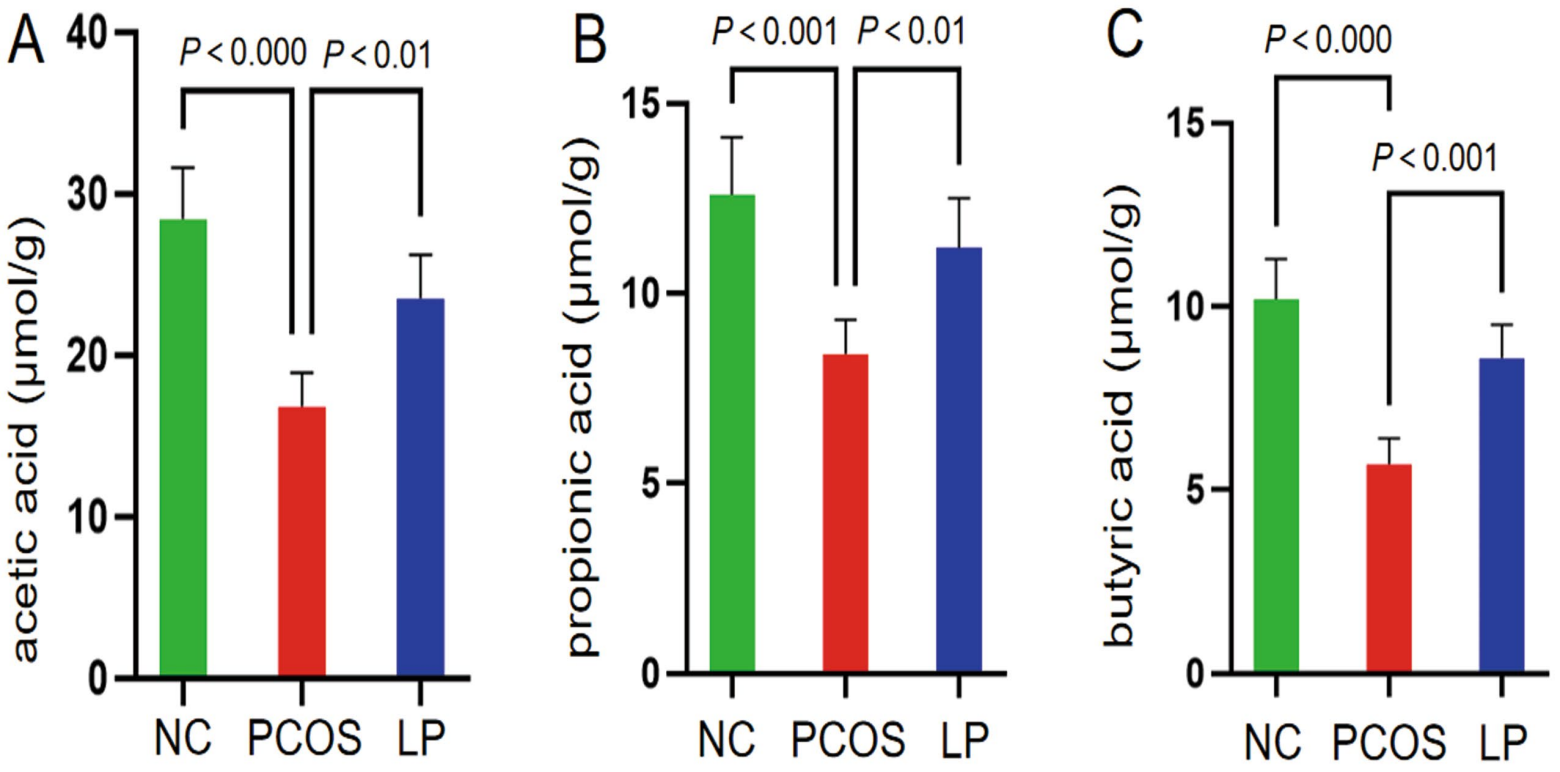
Quantitative analysis of short-chain fatty acid (SCFA) concentrations (μmol/g) in intestinal contents (*n* = 6). Panels illustrate measured levels of **(A)** acetic acid, **(B)** propionic acid, and **(C)** butyric acid.

Concurrent UPLC-MS/MS quantification of fecal SCFAs demonstrated that PCOS mice exhibited markedly reduced acetate, propionate, and butyrate levels relative to NC controls (*p* < 0.05), whereas LP treatment significantly upregulated all three SCFAs (*p* < 0.05), with particularly pronounced effects on butyrate recovery (Figure 6). These findings collectively suggest that the high-fat diet/letrozole-induced PCOS model induces both microbial dysbiosis and SCFA depletion, while LP administration effectively counteracts these metabolic disturbances.

### Influence of LP on endogenous serum metabolites in PCOS mice

**Figure 7:**
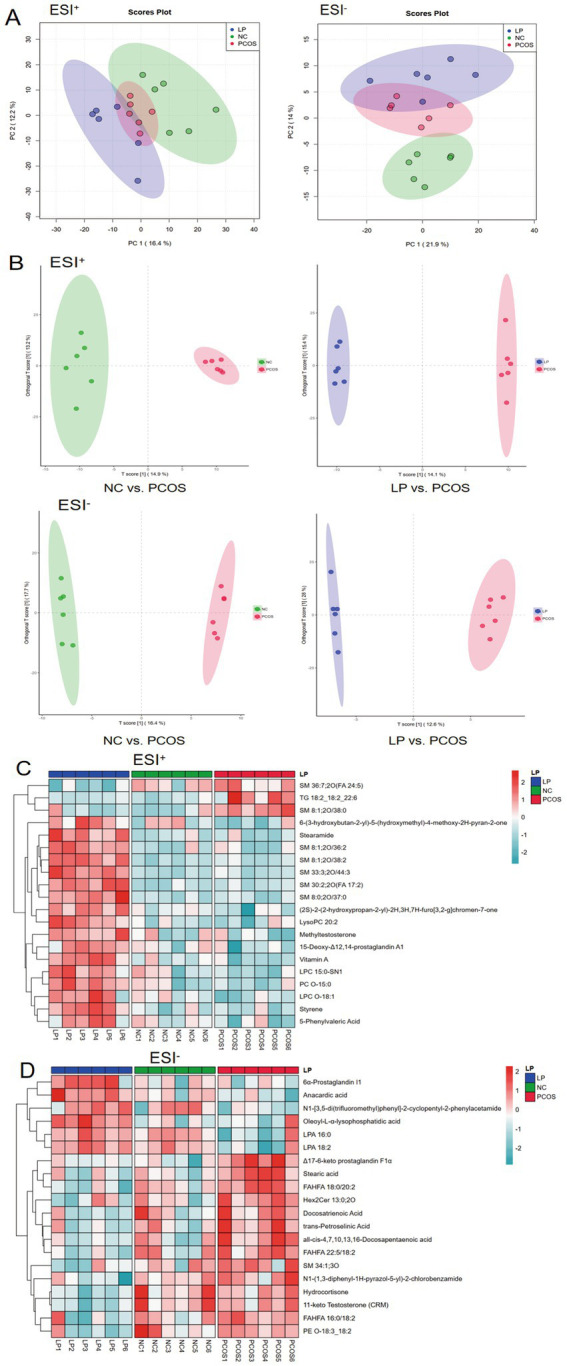
Serum metabolomics profiling and differential metabolite analysis (*n* = 6). Multivariate statistical analyses display: **(A)** Principal component analysis (PCA) score plot demonstrating group separation, **(B)** orthogonal projections to latent structures discriminant analysis (OPLS-DA) model visualization, with subsequent hierarchical clustering of significantly altered metabolites shown in heatmaps for **(C)** ESI positive and **(D)** ESI negative ionization modes.

Untargeted serum metabolomics revealed distinct metabolic profiles among the NC, PCOS, and LP-treated groups. Unsupervised PCA analysis demonstrated clear model interpretability, with evident segregation of sample clusters in both ESI + and ESI- modes. Specifically, the PCOS group was distinctly separated from the NC group, confirming successful model establishment. Notably, the LP-treated group exhibited a metabolic profile that shifted away from the PCOS cluster and toward the NC group, visually indicating the restorative effect of LP treatment (Figure 6A). To enhance predictive capacity and identify key metabolites, we performed OPLS-DA modeling. The model showed clear group separation (Figure 6B) and was rigorously validated. Differential metabolites (VIP > 1, *p* < 0.05) identified by OPLS-DA were subsequently visualized in hierarchically-clustered heatmaps (Figures 6C,D), further detailing the metabolic alterations. LP administration upregulated lipid mediators including SM33:3;20/44:3, LysoPC 20:2, and oleoyl-L-*α*-lysophosphatidic acid while downregulating SM 8:1;20/38:2 series. Anti-inflammatory effects were evidenced by elevated 6α-prostaglandin 11 with concurrent reductions in Δ17-6-keto prostaglandin F1α and docosatrienoic acid. Hormonal modulation involved increased pyranone derivatives [(2S)-2-(2-hydroxypropan-2-yl)-2H,3H,7H-furo[3,2-g]chromen-7-one] alongside decreased hydrocortisone and 11-ketotestosterone (Figure 7).

### Influence of LP treatment on metabolic pathways in PCOS mice

**Figure 8:**
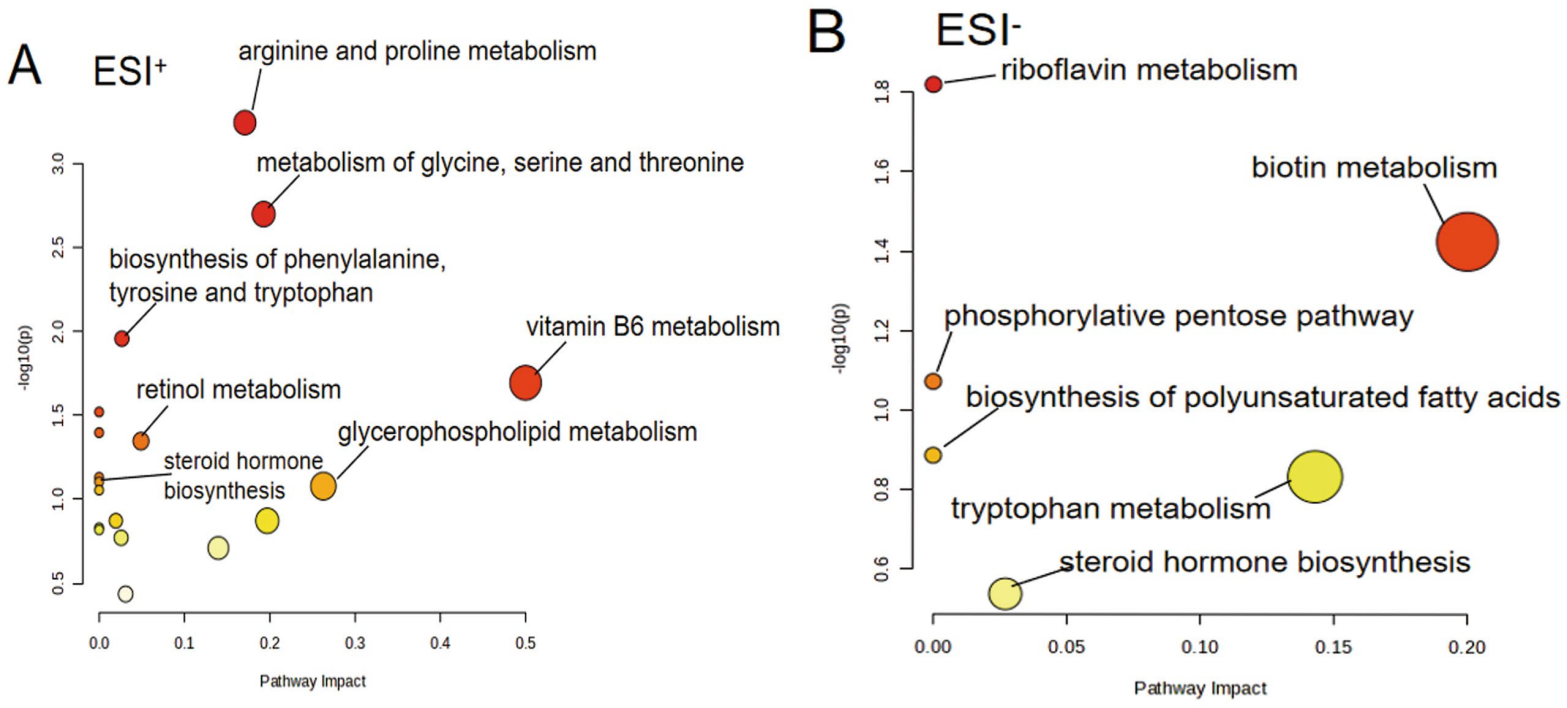
Metabolic pathway analysis of serum samples showing LP-mediated modulation in PCOS mice under **(A)** positive and **(B)** negative electrospray ionization modes (*n* = 6).

Differential metabolites were subjected to pathway analysis using MetaboAnalyst 5.0, with visualization displaying pathway impact values (x-axis) versus negative log-transformed *p*-values (y-axis). The enrichment results demonstrated LP’s significant regulatory effects on multiple interconnected metabolic pathways in PCOS mice, particularly involving amino acid metabolism, glycerophospholipid biosynthesis, vitamin metabolism, and steroid hormone synthesis (Figure 8).

## Discussion

This study systematically investigated the therapeutic potential and mechanistic basis of LP intervention in a PCOS murine model. The results revealed a significant effect of LP administration on the key pathological features of PCOS. Specifically, it was found that LP ameliorated metabolic dysfunction, corrected sex hormone imbalance, and alleviated ovarian pathological alterations. Furthermore, mechanistically, it was demonstrated that the therapeutic effects of LP were linked to its ability to restore gut microbiota homeostasis, enhance the production of short-chain fatty acids, and optimize the host’s systemic metabolic network.

The current study utilized *L. plantarum* NKK20 strain, isolated from healthy human fecal samples, which exhibits superior butyrogenic capability (21). Extensive evidence suggests that butyrate, as a pivotal gut microbial metabolite, orchestrates host metabolic regulation through two distinct pathways - modulating insulin sensitivity *via* GPR41/43 receptor activation while concurrently regulating epigenetic modifications through histone deacetylase (HDAC) inhibition (30–32). Our experimental data demonstrated that LP intervention not only elevated intestinal butyrate levels in PCOS mice but also coordinated the reduction of serum testosterone (T), luteinizing hormone (LH), and LH/FSH ratio, accompanied by significant downregulation of ovarian inflammatory mediators, revealing the compound’s potent immunomodulatory properties. Mechanistically, butyrate functions as a natural ligand for peroxisome proliferator-activated receptor *γ* (PPARγ) (33), suppressing NF-κB signaling pathway to attenuate ovarian expression of pro-inflammatory cytokines including TNF-*α*, IL-6, IL-1β and MCP-1 (34, 35), while simultaneously potentiating regulatory T cell (Treg) differentiation and IL-10 secretion - findings corroborated by our observation of significantly enhanced IL-10 expression in ovarian tissues following LP administration (36, 37). Furthermore, LP treatment upregulated intestinal tight junction proteins (ZO-1 and Occludin), reinforced gut barrier integrity, and reduced serum endotoxin levels (38, 39), collectively ameliorating PCOS-associated metabolic endotoxemia (40, 41). These comprehensive results delineate a “gut microbiota-butyrate-ovarian inflammation” regulatory cascade that fundamentally underpins LP’s therapeutic efficacy against hyperandrogenism in PCOS pathogenesis.

These findings, together with the present study, delineate a “gut microbiota-butyrate-ovarian inflammation” regulatory cascade that fundamentally underpins LP’s therapeutic efficacy against hyperandrogenism in PCOS pathogenesis. This proposed multi-tissue pathway integrates insights from existing research on gut-brain-ovary axis communication and metabolite-mediated immunomodulation, thereby providing a mechanistic framework that extends beyond isolated metabolic or hormonal corrections. The strength of this study lies in the systemic validation of this cascade, from microbial shift (increased butyrate) to molecular targets (PPARγ/NF-κB/Tregs) and finally to pathological improvement (hyperandrogenism, inflammation). However, the translational implications should be interpreted with consideration of its limitations. The study was conducted in a preclinical murine model, and although butyrate’s role in humans is supported by epidemiological and interventional studies (e.g., 42, 43), the therapeutic efficacy and safety of the specific NKK20 strain in human PCOS require further clinical validation. Additionally, while our data emphasize butyrate, other microbial metabolites or strain-specific effects could contribute to the observed outcomes, warranting future metabolomic and multi-omics investigations to fully disentangle the causal network. Lastly, the interplay between butyrate, other SCFAs, and host genetics in modulating PCOS phenotype remains an open question for personalized therapeutic strategies.

The metabolomics analysis revealed a particularly noteworthy negative correlation between butyrate levels and both serum testosterone concentrations and ovarian pro-inflammatory cytokines, suggesting butyrate may serve as the pivotal mediator linking gut microbiota alterations to core PCOS pathologies (18, 42). Previous reports of reduced butyrate-producing bacteria in PCOS patients were supported by our observation that LP intervention significantly increased the abundance of SCFA-producing genera like *Muribaculum* (43–45). However, while LP treatment robustly elevated intestinal SCFA levels (particularly butyrate), 16S rRNA sequencing demonstrated statistically insignificant changes in other established SCFA producers (*Faecalibacterium*, *Roseburia*, *Eubacterium*) (46, 47), likely reflecting inherent methodological constraints: although powerful for microbial profiling, 16S rRNA sequencing offers limited taxonomic resolution and cannot reliably determine functional metabolic activity (48, 49). Additional technical limitations including PCR amplification bias and sequencing depth may further obscure detection of low-abundance SCFA producers (50, 51). These considerations warrant future investigations employing metagenomic sequencing to achieve higher-resolution functional characterization of microbial communities (52, 53).

Untargeted serum metabolomics analysis demonstrated that LP administration profoundly modulated metabolic profiles of polyunsaturated fatty acids and sphingolipids, with elevated concentrations of specific lysophosphatidylcholine (LPC) species, including LysoPC 20:2 - an arachidonic acid-derived metabolite previously associated with improved insulin sensitivity (54, 55). This bioactive lipid mediates anti-inflammatory effects through PPARγ-mediated suppression of NF-κB signaling, mirroring our observation of elevated LPC O-15:0 and LPC O-18:1 (56–58). The former acts as a key precursor for plasmalogen biosynthesis (endogenous antioxidants) (59, 60), while the latter, an ether-linked phospholipid, contributes to membrane repair and oxidative stress mitigation (61, 62). We postulate that LP-induced butyrate enrichment triggers PPARα activation to upregulate ether lipid synthases (FAR1, AGPS), thereby enhancing production of these cytoprotective lipids (63, 64). Furthermore, LP-mediated elevation of intestinal short-chain fatty acids provides propionate as a potential substrate for LPC 15:0-SN1 biosynthesis, establishing a plausible microbial-lipid axis underlying metabolic improvement in PCOS (65, 66).

Therapeutic administration of LP directly modulated androgen homeostasis by altering key intermediates in steroidogenesis, most notably suppressing the generation of 11-ketotestosterone (11-KT) – a potent bioactive androgen in rodents that mirrors clinical hyperandrogenemia in PCOS patients (67–69). This reduction likely occurs through multiple coordinated mechanisms: (1) enzymatic inhibition of 11*β*-hydroxysteroid dehydrogenase (11β-HSD1) activity, curtailing the conversion of testosterone to 11-KT (70); (2) upregulated expression of hepatic 3α/β-hydroxysteroid dehydrogenases (3α/β-HSD) that catalyze the inactivation and subsequent excretion of testosterone and 11-KT into androstenedione and etiocholanolone (71, 72); (3) improvement of systemic insulin sensitivity in adipose tissue, thereby reducing insulin resistance-driven overexpression of CYP17A1 and subsequent testosterone overproduction (73); and (4) attenuation of metabolic endotoxemia by restoring gut barrier integrity, consequently decreasing LPS translocation and TLR4/NF-κB-mediated ovarian inflammation that promotes local androgen synthesis (74).

Notably, LP intervention significantly attenuated serum hydrocortisone levels in PCOS mice—a phenomenon potentially attributable to the coordinated modulation of hypothalamic–pituitary–adrenal (HPA) axis hyperactivity commonly observed in insulin-resistant states (75). Elevated circulating cortisol in PCOS may result from hepatic 11β-HSD2 suppression (reducing cortisol-to-cortisone inactivation), chronic low-grade inflammation, and adrenal overactivation (76, 77). We propose that LP exerts multimodal hydrocortisone-lowering effects through: (1) gut-brain axis-mediated downregulation of hypothalamic corticotropin-releasing hormone (CRH), consequently decreasing ACTH-driven adrenal cortisol production; (2) enhanced systemic insulin sensitivity that restores hepatic 11β-HSD2 activity, facilitating cortisol inactivation while simultaneously mitigating visceral adipose tissue inflammation and associated local hydrocortisone synthesis; and (3) improved gut barrier function that reduces endotoxin-mediated TNF-*α*/IL-1β stimulation of the HPA axis, complemented by propionate-mediated GPR43 signaling that directly suppresses adrenal hydrocortisone hypersecretion.

At the inflammatory level, LP treatment elicited a marked suppression of pro-inflammatory cytokines (TNF-α, IL-1β, IL-6) alongside augmented IL-10 expression in ovarian tissue—changes that correlated strongly with alterations in specific prostaglandin metabolites in systemic circulation. Particularly noteworthy was the modulation of 6a-PGI1, a 6α-hydroxylated derivative of the anti-inflammatory eicosanoid PGI₂ that arises during COX/PGIS-mediated enzymatic hydroxylation of arachidonic acid (AA) (78). This metabolite exerts pleiotropic benefits through NF-κB pathway inhibition, ultimately mitigating tissue inflammation while improving adipose insulin sensitivity (79). Mechanistically, LP may facilitate 6a-PGI1 biosynthesis via two complementary pathways: (1) secretion of PLA₂-activating factors that enhance AA liberation from intestinal epithelial membranes, thereby increasing PGI₂ precursor availability (80), and (2) NF-κB suppression by SCFAs that potentiates COX activity to further drive 6a-PGI1 production (81, 82). Concurrently, treatment significantly reduced levels of △17-6-keto PGF₁α—a pathogenic metabolite generated from PGF₂α, which contributes to ovarian fibrosis and insulin resistance—through SCFA-mediated NF-κB/PPARγ-dependent downregulation of COX (83, 84). These coordinated modulations suggest that LP’s immunoregulatory effects in PCOS operate via an integrated mechanism involving both direct SCFA-mediated immunomodulation (through dendritic cell/Treg regulation) and indirect prostaglandin pathway remodeling.

Notably, our findings reveal that LP supplementation significantly elevated circulating vitamin A levels in PCOS mice—a metabolic alteration with multifaceted therapeutic implications. Mechanistically, vitamin A exerts protective effects by attenuating ovarian ROS generation, thereby ameliorating oxidative stress and inflammatory damage, while concurrently suppressing testicular androgen biosynthesis through inhibition of CYP17A1 enzymatic activity, ultimately reducing serum free testosterone concentrations (85–87). These observations collectively suggest that LP may function as a vitamin A sensitizer, potentially explaining its efficacy in alleviating hallmark PCOS symptoms. The observed vitamin A deficiency typically associated with PCOS could originate from diet-induced chronic inflammation impairing intestinal absorption (88), a phenomenon potentially reversible through LP’s dual mechanism: (1) restoration of gut barrier integrity to minimize LPS translocation and subsequent systemic inflammation (89), and (2) direct enhancement of vitamin A bioavailability through microbial metabolites that facilitate enterocyte uptake (90).

LP intervention demonstrates superior therapeutic potential compared to current PCOS management strategies. While conventional approaches like metformin primarily target insulin resistance with minimal impact on gut microbiota modulation (91), and oral contraceptives effectively reduce hyperandrogenism at the expense of adverse metabolic effects (92), our study reveals that LP simultaneously addresses the triad of PCOS pathogenesis – metabolic dysfunction, hormonal imbalance, and chronic low-grade inflammation. This multi-pronged therapeutic efficacy highlights the unique advantage of microbial intervention through coordinated modulation of interconnected pathological pathways.

Several study limitations warrant consideration. The inherent biological discrepancies between rodent PCOS models and the heterogeneous clinical manifestations in human patients may affect translational relevance. Furthermore, although we observed significant alterations in SCFA profiles, the tissue-specific bioactivity of these microbial metabolites and their mechanistic association with discrete bacterial taxa require additional mechanistic validation. Dose–response relationships for LP intervention also remain to be systematically characterized. Future investigations should prioritize clinical translation, particularly examining strain colonization kinetics and interindividual variability in therapeutic responses among PCOS patients.

## Conclusion

Our investigation provides comprehensive evidence that *Lactiplantibacillus plantarum* NKK20 (LP) confers significant protection against PCOS pathogenesis by systemically reprogramming the gut-microbiota-metabolite-immune axis. The therapeutic efficacy of LP was evidenced by the amelioration of hallmark PCOS features, including insulin resistance, dyslipidemia, hyperandrogenism (e.g., reduced testosterone and 11-ketotestosterone), and the restoration of ovarian folliculogenesis.

The mechanistic insights gleaned from this multi-omics study reveal that LP’s actions are multifaceted. First, it attenuated gut dysbiosis and significantly elevated levels of SCFAs, with butyrate emerging as a pivotal mediator. This butyrate surge was associated with the reinforcement of the intestinal barrier (upregulation of ZO-1 and Occludin), reduction in endotoxemia, and subsequent suppression of ovarian NF-κB-driven inflammation (TNF-*α*, IL-6, IL-1β). Second, serum metabolomics uncovered LP’s profound impact on host metabolism, including the upregulation of insulin-sensitizing lysophosphatidylcholines (e.g., LysoPC20:2) and anti-inflammatory lipid mediators like 6α-prostaglandin I1, while downregulating pro-fibrotic metabolites such as Δ17-6-ketoprostaglandin F1α. The modulation of steroidogenesis and vitamin A metabolism further illustrates the hormonal and metabolic refinements induced by LP.

These findings substantially advance the concept of the gut-ovary axis, moving from correlation to causal mechanism. They position LP not merely as a probiotic but as a potent regulator of a complex host-microbial metabolic network. While this preclinical model offers compelling evidence, translation to the heterogeneous human PCOS population requires further clinical validation. Future research should focus on dose optimization, individual response variability, and the precise delineation of strain-specific mechanisms. Ultimately, this work lays a solid foundation for microbiota-directed interventions, positioning LP supplementation as a promising adjunctive strategy in the multidimensional management of PCOS.

## Data Availability

The original contributions presented in the study are publicly available. This data can be found here: https://ngdc.cncb.ac.cn/gsa/browse/CRA036093.
