# Supplementary material for: Multi-omics elucidation of Lactiplantibacillus plantarum NKK20 in preventing PCOS via the gut-ovary axis: SCFAs-mediated microbiota-metabolite-immune crosstalk
Source: Front Nutr. 2026 Jan 15;12:1709581. doi: 10.3389/fnut.2025.1709581 (PMC12853653; doi:10.3389/fnut.2025.1709581)
Supplement: Supplementary file 1 [file Table_1.docx]

**1. Explicit PCOS validation criteria:** The successful induction of PCOS phenotype was pathologically confirmed by daily vaginal smear examinations with the following diagnostic criteria: (1) persistent cornification (≥80% nucleated or keratinized epithelial cells observed for >10 consecutive days), (2) complete absence of leukocytic infiltration throughout the monitoring period, (3) disruption of normal estrous cyclicity characterized by the lack of progression through proestrus/estrus/metestrus/diestrus phases, and (4) maintenance of an extended anovulatory state (corresponding to constant estrus phase in rodents). These cytological findings collectively demonstrate both hyperandrogenism (through sustained vaginal epithelial cornification) and oligo-anovulation (via arrested cycle progression), which are pathognomonic features of PCOS rodent models.

2. Table S1 **Primer sequence and qPCR reaction system for inflammatory factor detection**

| Genes | Forward (5' → 3') | Reverse(5' → 3') |
| --- | --- | --- |
| *GAPDH* | AGGTCGGTGTGAACGGATTTG | TGTAGACCATGTAGTTGAGGTCA |
| *TNF-α* | CATCTTCTCAAAATTCGAGTGACAA | TGGGAGTAGACAAGGTACAACCC |
| *IL-6* | TAGTCCTTCCTACCCCAATTTCC | TTGGTCCTTAGCCACTCCTTC |
| *IL-1β* | GCAACTGTTCCTGAACTCAACT | ATCTTTTGGGGTCCGTCAACT |
| *MCP-1* | TTAAAAACCTGGATCGGAACCAA | GCATTAGCTTCAGATTTACGGGT |
| *IL-10* | GCTCTTACTGACTGGCATGAG | CGCAGCTCTAGGAGCATGTG |
| qPCR reaction system | | |
| SYBR Green Master Mix | | 10 μL |
| Forward/Reverse Primers (10 μM) | | 0.4 μL |
| cDNA Template | | 2 μL |
| ddH₂O | | 7.2 μL |
